# The role of personality variation, plasticity and social facilitation in cockroach aggregation

**DOI:** 10.1242/bio.036582

**Published:** 2018-12-15

**Authors:** Isaac Planas-Sitjà, Jean-Louis Deneubourg

**Affiliations:** Biological and Artificial Self-organised Systems Team - CP231, Université libre de Bruxelles (ULB), Campus Plaine, Bd. du Triomphe, Building No. 5, 1050 Brussels, Belgium

**Keywords:** Animal personality, Plasticity, Social facilitation, Collective behaviour, Insects, Cockroaches

## Abstract

Personality variation has been proven to affect ecology, evolution and group behaviour in many ways. Nevertheless, how social context influences behavioural strategies and individual personality variation has rarely been addressed. This study sheds light on the relationship between social interactions, personality variation and plasticity in a collective context. For this purpose, we used a binary setup (i.e. an arena with two identical shelters) to study the aggregation process of cockroaches. We tested the same individuals in isolated and social (groups of 16 individuals) conditions. We show that even if social interactions reduce the observation of personality variation, the behaviour in a group is correlated to individual preferences displayed in isolation. Furthermore, our results suggest that individuals show different levels of plasticity according to their shelter occupancy; individuals with high occupancy rates show low levels of plasticity and are less affected by social amplification in social conditions.

## Abstract (precis)

**Summary:** We used the American cockroach to show that individuals displaying low behavioural plasticity entrain the behaviour of other individuals through social interactions and change the aggregation dynamics of the group.

## INTRODUCTION

Animal personality has been defined as the existence of substantial variation in behavioural traits amongst individuals of the same population, with these differences being consistent within individuals over contexts or time (Dall and Griffith, 2014). When studying the behaviour of group-living animals, it is expected that the formation of a group reduces the individual differences within it (Krause and Ruxton, 2002). Different processes have been described to explain how individuals within a group can converge to express the same behaviour, such as conformity or social facilitation. Social facilitation, for instance, occurs when the presence of group mates affects the behaviour of an individual and allows, or causes individuals to engage in certain behaviours at a different rate, or to perform behaviours that they would not perform at all if they were alone (Zajonc, 1965). Therefore, social facilitation may affect the ways in which individuals within groups express personality traits in a number of different ways, adding a further layer of complexity (Webster and Ward, 2011). Despite widespread interest in animal personality variation on the one hand and in social effects (such as social organisation, social learning and anti-predator behaviour) on the other, to date, only a few investigations have addressed the issue of the responses of the same individuals in solitary and social contexts (e.g. Dyer et al., 2009; Jolles et al., 2016; McDonald et al., 2016; Van Oers et al., 2005; Webster et al., 2007).

In this study, we use the American cockroach (*Periplaneta americana*) to determine whether social influence produces a predictable, directional response in individual behaviour. For this purpose, we compared individual behaviour when the animals were tested alone and free of social influence with their behaviour when they were in a group. Recent studies have shown the existence of personality variation in their shelter-use behaviour of this species with implications at the collective level (Planas-Sitjà et al., 2015). Here, we test whether all individuals might increase their behaviour by the same degree or whether individuals might be affected differently; some individuals affected to a lesser or greater degree might entrain the behaviour of others and therefore have a disproportionate effect on the aggregation dynamics (Webster and Ward, 2011).

## RESULTS

### Isolated condition

To test the existence of behavioural differences between isolated individuals, we compared the experimental survival curves of the individuals' time bouts spent outside shelters. Isolated individuals had significantly different survival curves (see Table 1 and Fig. S2), meaning that their probability of visiting a shelter was significantly different.

**Table 1.**
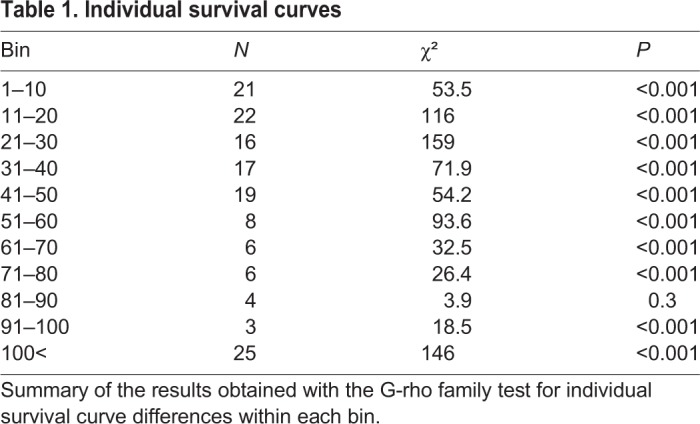
**Individual survival curves**

### Social condition

**Fig. 1.**
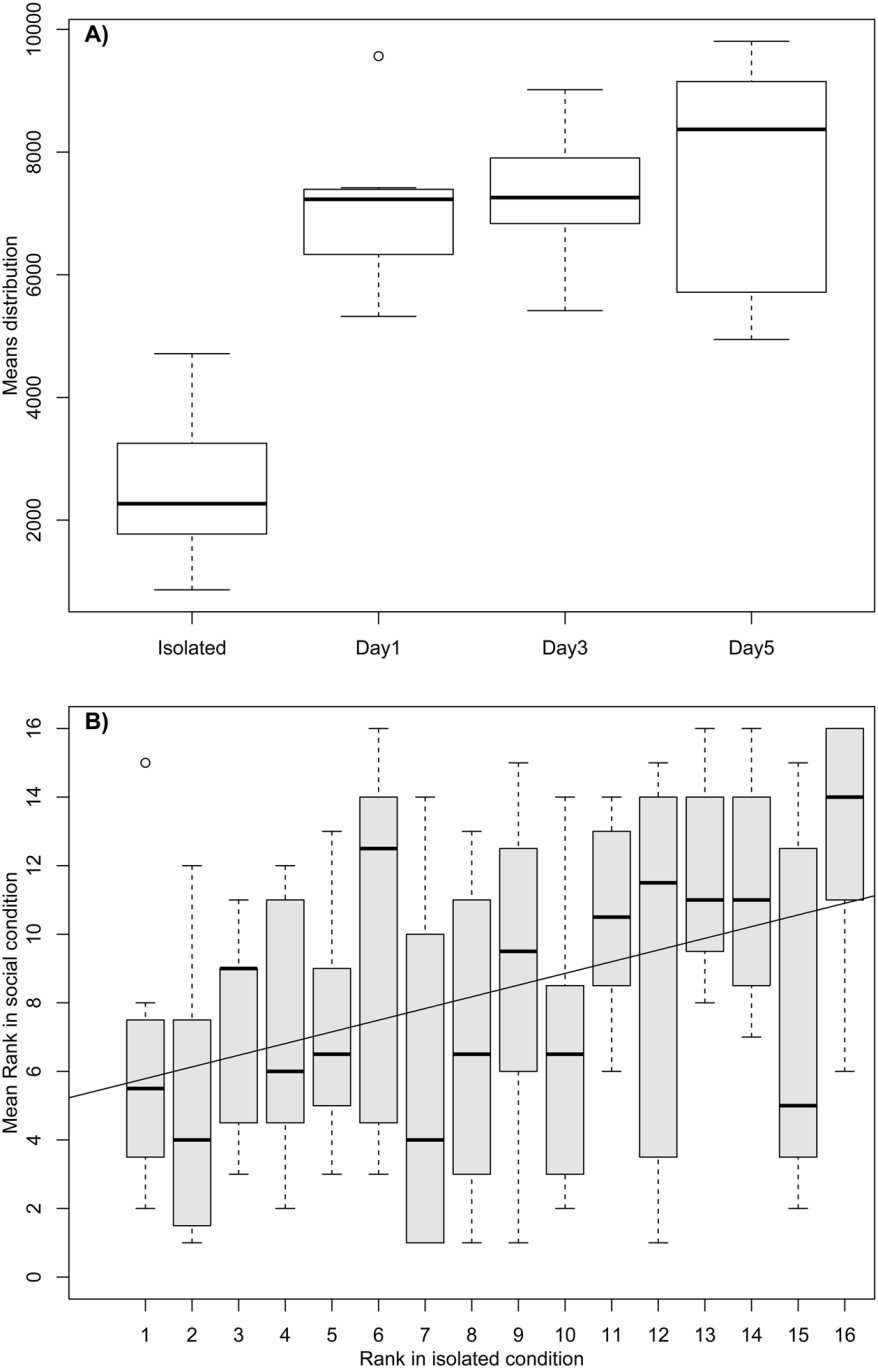
**Comparison or IRT between isolated and social conditions.** (A) IRT distribution: boxplots show the IRT distribution for individuals in isolated and social (day 1, day 3 and day 5) conditions. (B) Ranking relationship: boxplots show the distribution of the mean ranking (3 days) in social conditions and for each individual ranking in isolated conditions. Each boxplot is composed of eight points, one point per group. The black line is the linear regression between both rankings.

To analyse the effect of social interactions on individual behaviour, we compared the distribution of the individual resting times (IRT) between the isolated and social conditions (3 days together). Individuals in a social condition spent more time sheltered (greater IRT) than when in an isolated condition (D=0.68, *P*<0.0001; Fig. 1A).

We selected the best LMM model according to the AIC (Table 2) to analyse the behaviour of the individuals over days in the social condition (without taking into account the data from the isolated condition). The best model was the one that controlled for individuals, which significantly decreased the AIC value (ΔAIC=91.841), and a model also controlling for group factor did not improve this model. The LMM shows that the trial day (day in Table 2) had no significant effect although the value is very close to the significance level, thus possibly indicating a tendency for the time under the shelters to increase over trials. A further analysis comparing the mean IRT of the groups [mean IRT (s) day 1=7237; day 3=7480; day 5=7660; *N*=8] over trials, showed that the distribution of mean IRT did not vary day after day (Kruskal–Wallis: H=0.67, d.f.=14, *P*=0.72). In addition, while some groups show a majority of individuals increasing their IRT between days, other groups show the opposite: a majority of individuals decreasing their IRT (see Table S1). These results suggest that if individuals suffered sensitization or learning, it was a weak effect.

**Table 2.**
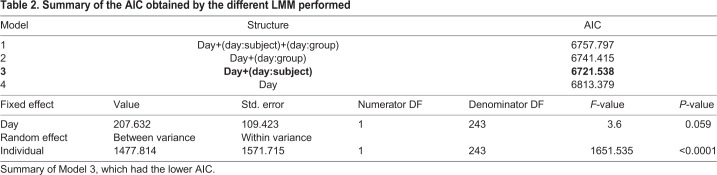
**Summary of the AIC obtained by the different LMM performed**

**Fig. 2.**
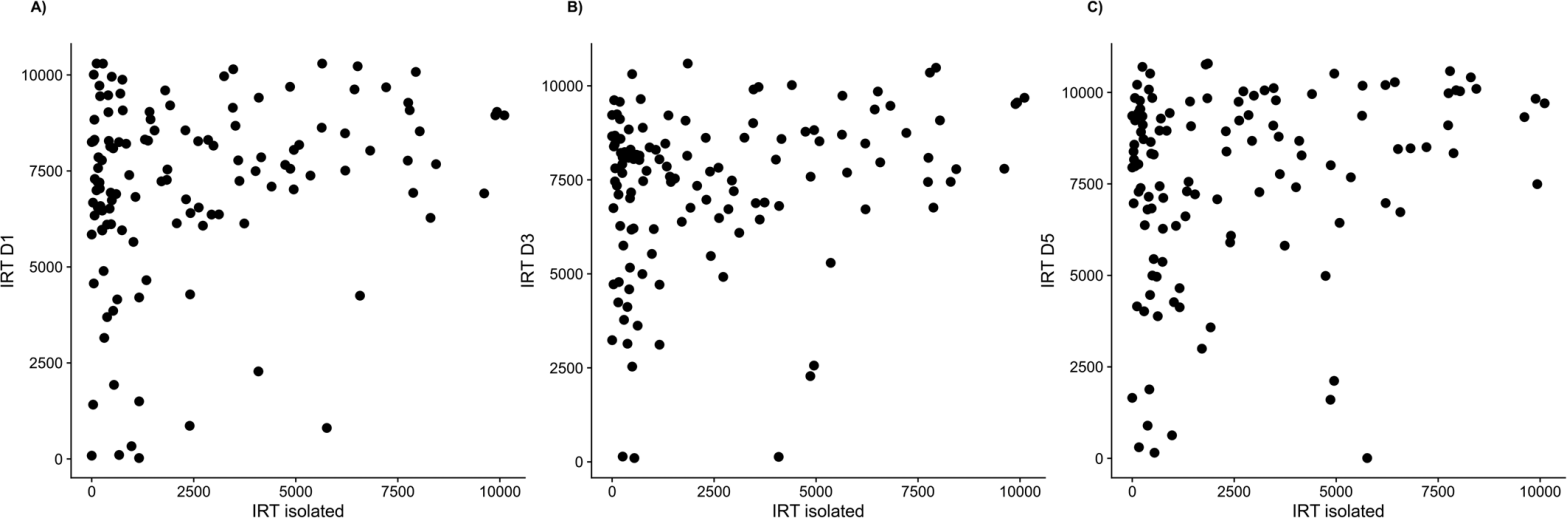
**IRT relationship.** Relation between IRT of experimental individuals in isolated and social conditions for (A) day 1 (IRT D1), (B) day 3 (IRT D3) and (C) day 5 (IRT D5).

In addition, the LMM shows an important influence of the individual; individuals had different slopes (Table 2; *P*<0.0001) and the time spent under shelters for each individual was more repeatable than expected (R=0.48, *P*<0.0001). In agreement with these results, we show a positive correlation between the individual ranking of IRT in the isolated condition and the mean rank (of 3 days) of the same individual in the social condition (linear regression: R^2^=0.12; *F*_1,122_=15.96, *P*=0.0001; see Fig. 1B). This result is in accordance with the interpretation of Fig. 2. Individuals with high IRT in an isolated condition keep a high IRT in social condition (points in top-right of the graphics and no points in the bottom-right; Fig. 2). Individuals with low IRT in isolated condition exhibited a range of social IRT (points in top-left and bottom-left).

**Fig. 3.**
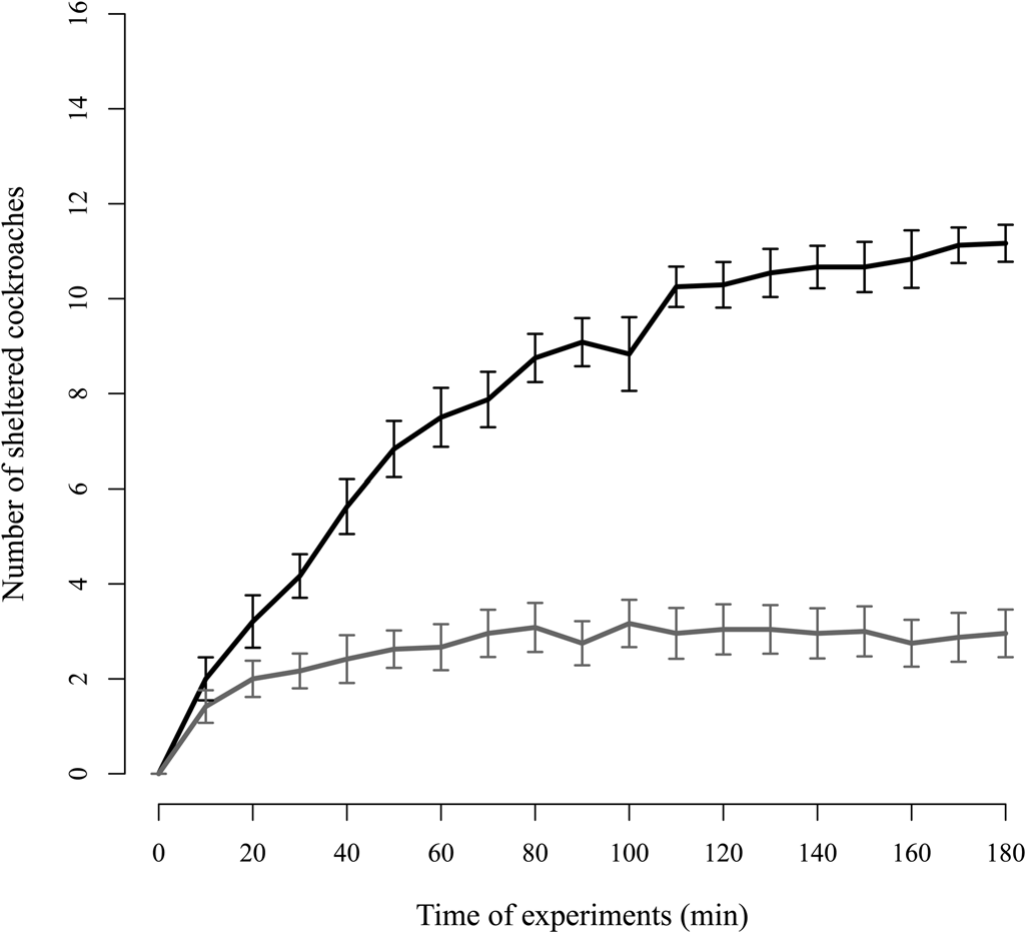
**Change in the number of sheltered cockroaches over the experiment.** Black line represents the mean number of cockroaches (±s.e.) present under the selected shelter (houses the majority of cockroaches at the end of the experiment) taking into account the 24 experiments. Grey line represents the non-selected shelter.

Finally, we analysed the change in the number of cockroaches aggregated under the shelters (Fig. 3). Interestingly, we observed that the choice of the aggregate is fast, and from minute 30 (approximately) we could already predict which shelter would house the majority of individuals (Fig. 3). Therefore, the first minutes of the aggregation are critical for the decision-making process.

## DISCUSSION

The aggregation process in cockroaches has been studied extensively (Amé et al., 2006; Canonge et al., 2011; Dambach and Goehlen, 1999; Jeanson et al., 2005; Pogson, 2016; Varadínová et al., 2010). Nevertheless, how social facilitation affects individual behaviour and plasticity has never been studied during the aggregation process. In this study we show that cockroaches increase their sheltering time when tested in a social condition, keeping similar individual rankings (regarding the IRT) over trials. These findings agree with previous studies showing that the probability of leaving a shelter decreases with the group size (Amé et al., 2006) and that individuals show personality variation when tested in a group (Planas-Sitjà et al., 2015). Importantly, we show that the rank of the IRT was in accordance with individual behaviour in an isolated condition (Fig. 1B); low IRT individuals increase or maintain their IRT in a social context (variable plasticity) while high IRT individuals, not surprisingly, keep a high IRT (low plasticity; see Fig. 2). Thus suggesting that some individuals show more behavioural plasticity than others when influenced by social interactions. A recent study on the cockroach *Blaberus discoidalis* showed that consistency of behaviour was maintained in a social condition, but not when the same individuals were tested alone, suggesting that individuals might vary in their social cohesion (Crall et al., 2015). In our study, we show a relationship between IRT rankings in isolated and social conditions. Our hypothesis is that *B. discoidalis* has a certain degree of hierarchy, as seen in other species of the same genera (Bell and Gorton, 1978; Gautier, 1974; Legendre et al., 2014), and therefore the social interactions within a group of *B. discoidalis* may be higher than in *P. americana*, which has a low and unstable degree of hierarchy (Bell and Sams, 1973).

These results altogether reopen the debate about the interplay between personality variation and self-organisation (Planas-Sitjà et al., 2015). During the aggregation process, *P. americana* forms groups in which all individuals do not have vastly different levels of influence on the final decision (Halloy et al., 2007). Here we show that, after approximately 30 min, we can already predict which shelter would be selected (Fig. 3). High IRT individuals are those settling earlier, if not, they could never reach IRT values around 10,000 s. Therefore, our hypothesis is that individuals that are more prompt to visit a shelter and remain under it (high IRT) may promote more aggregation than individuals that rarely visit the shelter. Thus, these individuals with a rapid settlement and that are less affected by social interactions, would be more likely to retain other individuals and exert a disproportionate effect on site aggregation and collective decision.

The question of a potential benefit from maintaining personalities within a group has been discussed by many authors (Dall and Griffith, 2014; Jandt et al., 2014; Jeanson and Weidenmüller, 2014; Jolles et al., 2018; Réale et al., 2007). Having personality variation within groups could be more efficient than having identical individuals with a high behavioural plasticity (Jandt et al., 2014). Indeed, hydrocarbons found on the body surface act as attractant for other individuals (e.g. Saïd et al., 2005), and therefore amplifying the aggregation process. We hypothesise that the maintenance of personalities within a group could be a benefit because it could reduce the need for hydrocarbons, which modulate social attraction. If hydrocarbon production can be reduced by some individuals, without harming the aggregation process, it means that more energy can be invested in other vital aspects, such as reproductive behaviour or foraging and therefore increases the individual’s fitness. Future studies investigating the relation between hydrocarbons and behavioural phenotype will be useful to shine light on these hypotheses.

In conclusion, we show that some phenotypes with low behavioural plasticity, asymmetrically affected by the social facilitation, could entrain the behaviour of other individuals with more behavioural plasticity and change the performance and settlement efficiency of the group.

## MATERIALS AND METHODS

### Experimental design

#### Biological model

*Periplaneta americana* (L.) (Dictyoptera: Blattidae) is a domiciliary cockroach that forms aggregates during daylight hours in dark and warm places and is active during night-time. The cockroaches used in this study measured from 35–50 mm in length and were issued from strains reared in breeding facilities (five Plexiglas vivaria of 80×40×100 cm) of the Université libre de Bruxelles (ULB). Each vivarium contained about 1000 individuals of both sexes and of all developmental stages and were provided with dog pellets and water twice a week. The rearing room was maintained at 25±1°C under a 12:12 h light/dark (L/D) cycle.

#### Experimental setup

Experiments were carried out on adult male without external damage (see Laurent Salazar et al., 2018; Planas-Sitjà et al., 2015 for more information). The experimental set-up was a circular arena, covered with a paper layer (120 g/m^2^), surrounded by a black polyethylene ring (diameter: 100 cm, height: 20 cm) with a light source (four General Electric Energy-saving light bulbs, 23 W, 2700 K) placed above the set-up. To prevent cockroaches from escaping, the inner surface of the ring was covered by an electric fence (Canonge et al., 2009). This fence was placed high enough (8 cm from the floor) to avoid disturbing the activity of cockroaches when being close to the wall. Two shelters made of transparent Plexiglas discs (diameter: 15 cm) with three Plexiglas feet (diameter: 0.2 cm) were covered by a red-coloured filter film (Rosco E-Colour 19: fire), creating low luminosity zones. Cockroaches are photophobic during the diurnal phase, so both shelters are perceived as resting sites (Bell and Adiyodi, 1982). The centre of each disc was located 23 cm from the edge of the arena and stood 3 cm above the floor arena. The set-up was surrounded by white tissue to avoid the inclusion of spatial cues (see Fig. S1 for more details).

To allow the detection of the animals when they were under the shelter, the cockroaches were tagged with an RFID chip (diameter: 7.1±0.2 mm, weight: 107±3 mg; Spacecode). This chip was glued to the thorax with Latex (Winsor & Newton). Sheltered individuals were detected by a circular RFID reader located below each shelter, which recorded the presence and identity of each individual sheltered (approximately every 3 s). From this data we calculated (a) the number of individuals inside each shelter, (b) the total amount of time spent inside/outside shelters and (c) the number of visits to each shelter (see Planas-Sitjà et al., 2015 for more details).

#### Experimental procedure

Groups of 16 cockroaches were kept in almost total darkness (∼ 70 l×) for 48 h in Plexiglas boxes (36×24×14 cm) containing a cardboard shelter, humidified cotton wool and *ad libitum* food. Then, one male was introduced in the centre of the arena. Because we had three identical setups in the same room, we could test three individuals at the same time, and each trial lasted 3 h. During this time, the animals were free to explore the arena and visit the shelters. This procedure was repeated twice a day (9–12 h and 14–17 h) for 3 days (Tuesday, Wednesday and Thursday), with a total of 16 cockroaches tested in isolated condition. After the trials, the cockroaches were introduced into a new Plexiglas box and kept in almost total darkness for 72 h. Then, the 16 males were introduced to the centre of the arena. The same group of cockroaches was tested during three consecutive trials (each trial lasted 3 h) over a week (Monday, Wednesday and Friday), with a 45-h gap between trials, where the groups were kept in the dark in the same Plexiglas box. This procedure was repeated for eight different groups. We measured the sheltering time (under both shelters) for each cockroach throughout the experiment. In this article, we refer to it as the IRT in isolated and social conditions. In social conditions, we also measured the total number of cockroach present under each shelter every 10 min.

### Statistical analysis

We used Python 3 (Python Software Foundation) for data treatment and R software (R Core Team, 2016, v. 3.3.2) for statistical analysis. We used the Kolmogorov–Smirnov test (KS test) to analyse the differences in shelter use between conditions (isolated or group). The linear model was used for the regression analysis of the time spent under shelters between trials. We used a comparison of survival curves of the individuals' time bouts spent outside shelters to assess behavioural differences in isolated conditions. As individuals showed high variability in the number of visits to shelters (ranging between 2–200), and some individuals did not perform enough visits to allow any analysis (e.g. 2–20 visits), we decided to divide our cockroaches depending on their number of visits to shelters with bins of 10 visits (e.g. 1–10, 11–20, 21–30, etc.). We did a histogram (Fig. S2) and we compared the survival curves of individuals within each range using the *G*^ρ^ family of tests (package ‘Survdiff’ in R).

We used a linear mixed model (LMM) to assess behavioural consistency between trials in social conditions and to test whether individuals where more prompt to shelter over trials as a result of light sensitization or learning the position of the shelters. The significance of the statistical tests was fixed to α=0.05.

## Supplementary Material

Supplementary information
