# Supplementary material for: The role of personality variation, plasticity and social facilitation in cockroach aggregation
Source: Biol Open. 2018 Dec 15;7(12):bio036582. doi: 10.1242/bio.036582 (PMC6310888; doi:10.1242/bio.036582)
Supplement: Supplementary information [file biolopen-7-036582-s1.pdf]

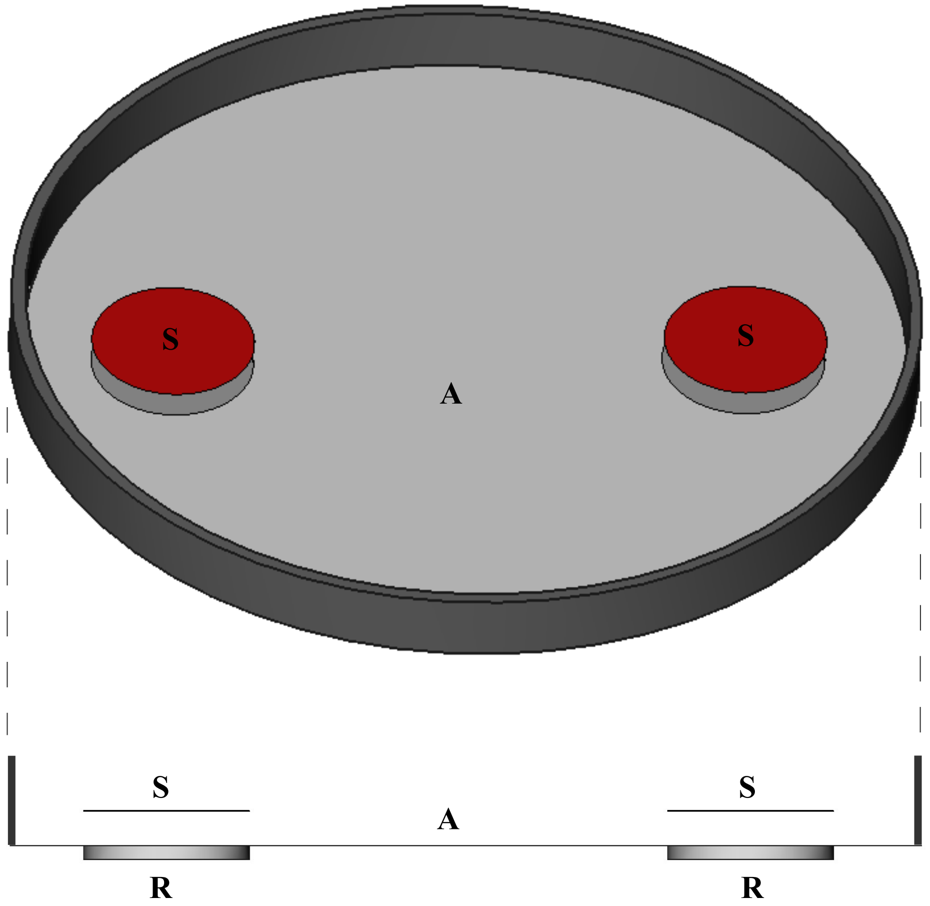

Fig. S1: Design of the experimental set-up in a perspective and lateral view. A: Arena of the set-up; S: Shelters with red filter; R: RFID reader.

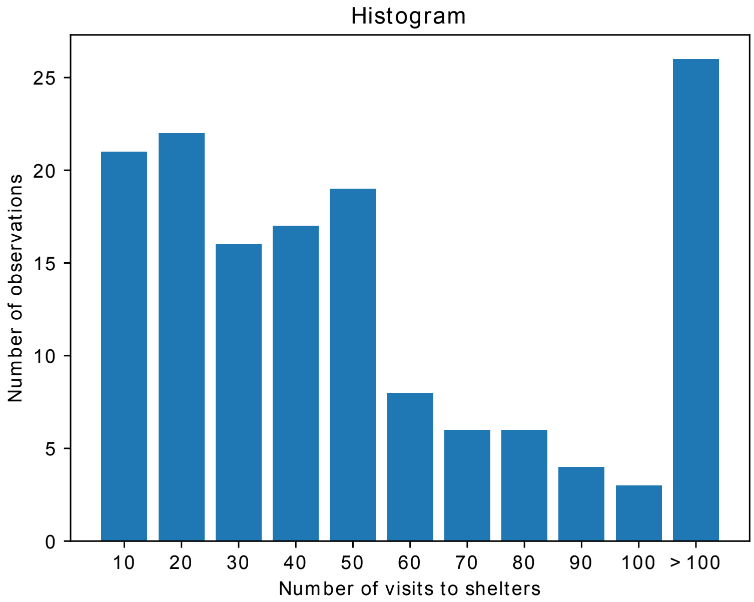

Fig. S2: Histogram showing the number of individuals depending on the number of visits to shelters. The histogram shows all the individuals tested. The >100 bar contains individuals spending from 101 s to10,000 s sheltered.

Biology Open • Supplementary information

Table S1. *Number of cockroaches decreasing or increasing their individual resting time (IRT) between trials. (*) Chi-squared test P-values<0.05*

		Group 1	*Group 2*	*Group 3*	*Group 4*	*Group 5*	*Group 6*	*Group 7*	*Group 8*
Isolate – Day 1	*Decrease*	2	3	0	0	2	4	0	2
	Increase	13*	13*	14*	15*	14*	11	14*	13*
Day 1 – Day 3	*Decrease*	1	9	8	10	8	6	5	10
	Increase	14*	7	6	5	8	9	9	5
Day 3 – Day 5	*Decrease*	2	1	5	9	13*	12	2	3
	Increase	13*	15*	9	6	3	3	12*	12

Biology Open • Supplementary information
